# A Comprehensive Comparison and Evaluation of AI-Powered Healthcare Mobile Applications’ Usability

**DOI:** 10.3390/healthcare13151829

**Published:** 2025-07-26

**Authors:** Hessah W. Alduhailan, Majed A. Alshamari, Heider A. M. Wahsheh

**Affiliations:** Department of Information Systems, College of Computer Science and Information Technology, King Faisal University, Al-Ahsa 31982, Saudi Arabia; 223002437@student.kfu.edu.sa (H.W.A.); hwahsheh@kfu.edu.sa (H.A.M.W.)

**Keywords:** Usable-AI, healthcare, usability evaluation, mHealth, artificial intelligence

## Abstract

**Objectives**: Artificial intelligence (AI) symptom-checker apps are proliferating, yet their everyday usability and transparency remain under-examined. This study provides a triangulated evaluation of three widely used AI-powered mHealth apps: ADA, Mediktor, and WebMD. **Methods**: Five usability experts applied a 13-item AI-specific heuristic checklist. In parallel, thirty lay users (18–65 years) completed five health-scenario tasks on each app, while task success, errors, completion time, and System Usability Scale (SUS) ratings were recorded. A repeated-measures ANOVA followed by paired-sample *t*-tests was conducted to compare SUS scores across the three applications. **Results**: The analysis revealed statistically significant differences in usability across the apps. ADA achieved a significantly higher mean SUS score than both Mediktor (*p* = 0.0004) and WebMD (*p* < 0.001), while Mediktor also outperformed WebMD (*p* = 0.0009). Common issues across all apps included vague AI outputs, limited feedback for input errors, and inconsistent navigation. Each application also failed key explainability heuristics, offering no confidence scores or interpretable rationales for AI-generated recommendations. **Conclusions**: Even highly rated AI mHealth apps display critical gaps in explainability and error handling. Embedding explainable AI (XAI) cues such as confidence indicators, input validation, and transparent justifications can enhance user trust, safety, and overall adoption in real-world healthcare contexts.

## 1. Introduction

Healthcare is undergoing a global transformation with the increasing adoption of artificial intelligence (AI) technologies. These advancements are revolutionizing patient care, diagnostics, treatment, and healthcare management workflows. AI subfields such as computer vision, natural language processing, machine learning, and deep learning have made significant contributions to the evolution of healthcare services [1]. While non-AI applications in healthcare are generally limited to basic functions such as symptom checking and appointment scheduling, AI-enabled healthcare applications provide advanced decision support, personalized care, and clinical insight based on data-driven algorithms [2].

Given that patients are the end users of these AI-powered systems, it is essential that such applications are designed with a strong emphasis on usability, safety, and privacy. AI systems should be easy to use and should enhance patient navigation and satisfaction with healthcare services [3]. Thus, assessing the usability of AI healthcare applications is a critical step toward ensuring their effective and safe implementation.

AI refers to technologies that can perform cognitive and physical tasks and make decisions without direct human intervention [4]. Its components of machine learning, neural networks, genetic algorithms, and pattern recognition support various medical processes. AI can exceed human limitations and enhance productivity, especially in human–computer interaction (HCI), where it improves interface design, user engagement, and system safety [5,6]. In healthcare, AI is commonly used in clinical support systems and robotic surgery, though many systems remain minimally usable, posing potential risks to patients [7,8]. mHealth applications supported by AI aim to improve care quality, cost-efficiency, and health system management [2]. Despite these advantages, some users remain hesitant about AI’s role in healthcare due to concerns related to usability, trust, and transparency [2].

While AI apps offer advantages in administrative tasks, diagnosis, treatment, and follow-up care [3], users still encounter usability challenges [9]. For example, voice-driven smart assistants often underperform on complex queries [9,10], and design failures in HCI have contributed to critical incidents such as self-driving vehicle accidents [9]. In healthcare, design issues such as poor navigation, limited error recovery, and lack of transparency can compromise trust and usability key elements in medical environments that demand precision and reliability [11].

This study offers a comprehensive usability evaluation of three leading AI-based mHealth applications using both expert and user-based assessments. The focus is on the usability of these tools rather than the internal effectiveness of the underlying AI algorithms. The findings aim to support developers and designers with practical recommendations to improve the usability and user experience of AI-powered mobile health apps.

Few studies have systematically assessed the usability of AI-powered mHealth apps [3,12,13,14,15], and fewer still have used integrated methods such as heuristic evaluation, user testing, and automated assessments [16,17,18]. This study fills that gap by offering a combined usability evaluation framework, emphasizing user-centered design, and broadening existing models that traditionally prioritize algorithmic performance over human experience. Regarding the growth of the mHealth market, the fact that over 325,000 apps were available in 2017 alone [19] underscores the need for improved usability standards. Despite advances in AI modeling, user-centered design remains underrepresented in development processes [20]. Prominent AI health apps like Woebot [21] and Babylon Health [22] exemplify innovation in digital health but face challenges due to varied user health and digital literacy [23]. Previous research has highlighted usability concerns such as confusing outputs and unexpected errors [24], reinforcing the importance of transparent and intuitive design. In the following sections, we review the current literature, define our research objectives, and present a triangulated methodology to evaluate the usability of three AI-powered mHealth applications.

## 2. Literature Review

The emergence of mobile health applications (mHealth apps) with artificial intelligence (AI) has great potential to transform how healthcare is accessed and delivered. These technologies promise enhanced personalization, clinical decision support, and broader access to care, especially in underserved regions [3]. However, for such benefits to be realized, rigorous usability evaluations are essential to ensure these systems are effective, safe, and accessible in practice. The following subsections present an overview of core usability constructs, explainability in AI, empirical evaluations of mHealth AI tools, and the resulting gaps this study seeks to address.

### 2.1. Usability Constructs in mHealth

ISO 9241-11 defines usability as the extent to which specified users can achieve specified goals with effectiveness, efficiency, and satisfaction within a defined context [25]. Further work has introduced learnability and memorability, emphasizing core usability metrics such as user satisfaction, error detection, ease of learning, and task completion speed [26,27]. In the context of AI-enabled mHealth, these constructs are still central. A recent study highlights satisfaction, efficiency, learnability, and error prevention as the most frequently emphasized usability dimensions for AI-based apps [3]. This underscores the continued relevance of traditional usability metrics in mHealth evaluation, although they may not fully address the unique demands introduced by AI systems.

### 2.2. Explainable AI and Trust

Explainable artificial intelligence (XAI) aims to make AI outputs and processes more interpretable, which is critical for user trust and safety [7]. Since DARPA introduced the formal concept of XAI in 2017 [28], research has extended to various domains, including healthcare, autonomous systems, and finance [29]. However, healthcare AI remains challenged by a lack of explainable models and interfaces. Despite technical advances, many AI applications do not adequately communicate reasoning processes to end users, limiting transparency and trust [30]. This concern is particularly acute in healthcare, where unintelligible AI-driven outputs may lead to frustration, reduced usage, or even distrust of the system. Studies have shown that explainability plays a pivotal role in enhancing user confidence and ensuring patient safety when interacting with AI systems in digital health [31,32,33]. These findings indicate that explainability is not just a technical concern but a core usability factor that must be addressed in mHealth design.

### 2.3. Empirical Evaluations (2021–2024)

Recent studies have attempted to measure the real-world usability and trust of AI-integrated health tools, using a range of evaluation methods including heuristic reviews, user testing, and survey-based assessments. These studies provide valuable context for the present work. One study evaluated the usability of an AI-enhanced mobile health application in rural areas of Pakistan. Using ISO 9241-based metrics and user interviews [25], it identified challenges in accessibility and decision logic, particularly among users with low digital literacy [13]. Another investigation analyzed various pandemic-related mHealth applications, applying usability testing methods to reveal navigation and feedback limitations during task completion. The study emphasized the importance of simplicity and transparency in emergency health contexts [14].

A separate analysis of user reviews for Wysa, an AI-driven mental health chatbot, highlighted both positive sentiment around engagement and persistent concerns with AI understanding and accuracy [15]. An expert-based evaluation applied Nielsen’s heuristics to assess the usability of telemedicine apps during COVID-19 in Saudi Arabia. It identified recurring issues in error prevention and consistency, reinforcing the need for robust interface design [16]. A survey of 486 smartwatch users in Bangladesh explored trust and satisfaction with AI-based health monitoring features, finding that convenience and service quality were key predictors of user satisfaction [18]. A clinical dashboard powered by AI for peripheral artery disease detection was evaluated, showing that while physicians found the interface helpful for EHR integration, they struggled with the interpretability of predictions [17]. Finally, a randomized trial on Woebot, an AI chatbot delivering cognitive–behavioral therapy, reported strong engagement but highlighted the need for clearer explanations of AI logic [21].

### 2.4. Gap Synthesis

Although a growing body of work has examined the usability of AI-powered health applications, most studies have considered just one dimension at a time. For example, several papers employ expert or heuristic inspection or satisfaction surveys without live users [3], while others focus solely on end-user performance metrics such as task completion time and satisfaction scores [12,18]. Clinician-facing dashboards have also been evaluated primarily for layout clarity rather than the transparency of the underlying models [17]. Research on explainability likewise highlights transparency shortcomings but seldom connects them directly to measurable usability outcomes [11,30]. Recent reviews therefore call for multi-method designs that triangulate expert evaluation, real-user testing, and formal XAI assessment to understand how transparency gaps influence trust, task success, and long-term engagement with AI systems [31,32,33]. Addressing these intertwined factors within a single study remains an open challenge, and this study seeks to meet that challenge by integrating heuristic, user-centered, and explainability-oriented methods in a unified evaluation of leading AI-enabled mHealth applications.

## 3. Research Objectives and Paper Structure

The purpose of this section is twofold: first, to outline the specific research objectives that guided the study; second, to provide an overview of the paper’s structure to support the reader’s understanding of the logical flow of the work. By clearly stating the goals of the study and the organization of its content, we aim to establish a transparent framework for the subsequent sections.

### 3.1. Research Objectives

This study aims to accomplish the following:

1. Review the existing literature on the usability of AI-based health applications.
2. Conduct a comprehensive usability evaluation using expert heuristics, user testing, and basic automated methods.
3. Provide practical recommendations to improve the usability of AI-powered mHealth applications.

### 3.2. Paper Structure

The remainder of the paper is organized as follows: Section 4 describes the materials and methods. Section 5 details the results. Section 6 discusses key findings and implications. Section 7 concludes the paper and offers suggestions for future research.

## 4. Materials and Methods

**Figure 1:**
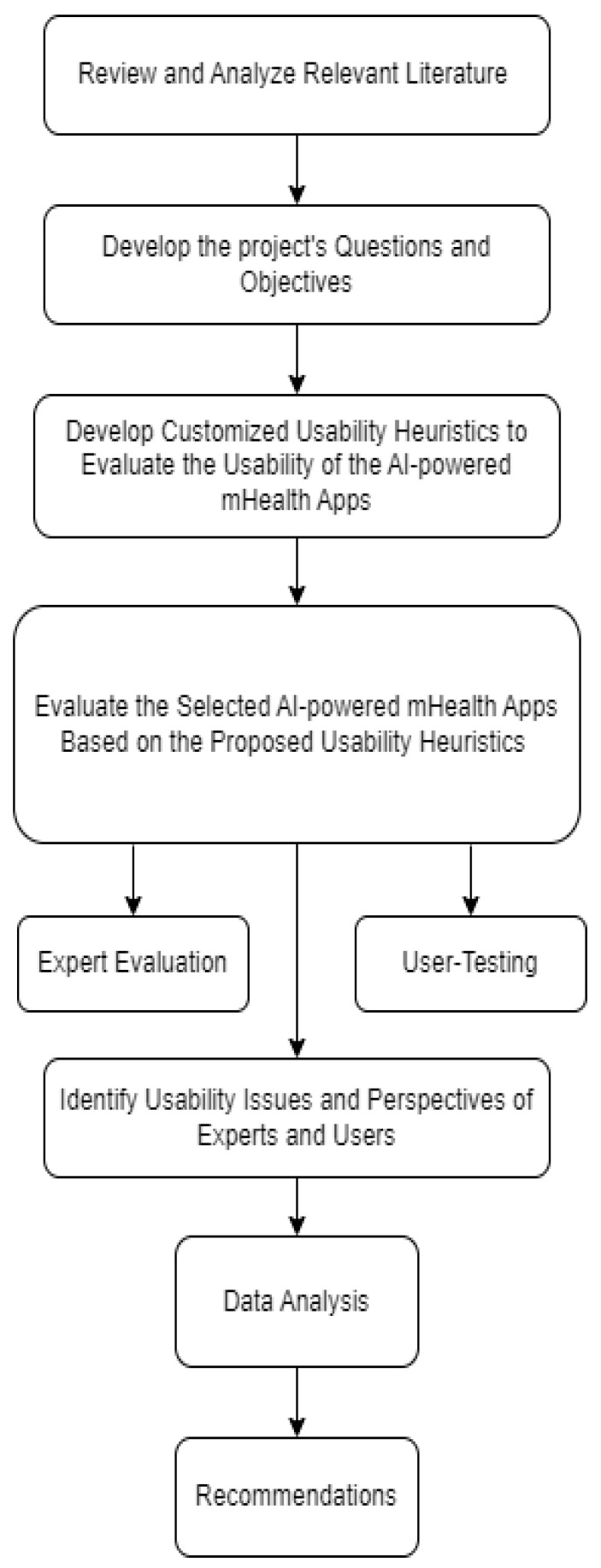
Research methodology.

To evaluate the usability of AI features in mHealth apps, this study adopts an experimental approach. Inspired by [13], which used ISO 9241-11 metrics [25], and [16], which applied Nielsen’s heuristics [26], this study follows a similar structure assessing effectiveness, efficiency, and satisfaction. The study begins with a literature review to shape its objectives and identify key evaluation factors related to usability and AI in mHealth. The evaluation approach comprises three phases: (1) a heuristic evaluation by experts using best practices; (2) user testing with tasks and think-aloud protocols; (3) automated usability tests to detect technical flaws and accessibility compliance. The methodology provides a comprehensive assessment of usability from both expert and user perspectives, culminating in actionable recommendations. Figure 1 illustrates the research methodology workflow.

### 4.1. Developing Customized Usability Heuristics

Heuristic evaluation, as introduced by [34], involves experienced evaluators assessing an interface against usability principles. Evaluators individually document usability issues and then consolidate their findings, ranking issues by severity, frequency, and criticality [35].

Table 1 lists Nielsen’s ten heuristics. Transparency and explainability are increasingly essential in AI design [36,37,38,39]. Users expect clear explanations and trustworthy systems [23,40,41].

Table 2 presents the proposed heuristics tailored to AI-powered mHealth apps.

### 4.2. App Selection Criteria

This study evaluated three widely used AI-powered mHealth applications [42]: ADA [43], Mediktor [44], and WebMD [45]. These applications were selected based on the following inclusion criteria to ensure relevance to real-world, general-use AI-based healthcare tools:

Popularity and Availability: Each app ranked among the most downloaded in the Health and Fitness or Medical categories on both the Apple App Store and Google Play Store in recent market analytics [42].

- User Ratings: Apps with a minimum average rating of 4.0 stars on both platforms were selected to ensure established user acceptance and quality [42].
- AI Integration: Apps were required to incorporate artificial intelligence features such as symptom checking, triage support, or diagnostic suggestions, aligning with the study’s focus on AI-powered healthcare usability.
- Language and Accessibility: Apps had to be available in English and provide core functionalities (e.g., symptom checker) without requiring paid subscriptions or institutional licensing, ensuring accessibility for general users.

### 4.3. Participant Recruitment and Selection

#### 4.3.1. User Participants

The study employed both inclusion and exclusion criteria to guide participant selection. A total of 30 participants (18 males, 12 females), aged between 18 and 65 years (mean = 33.4 years; standard deviation = 11.2), were recruited through purposive sampling to ensure diversity in health and digital literacy levels.

Inclusion Criteria:

- Aged 18–65 years.
- Regular users of mobile health or wellness apps.
- No prior experience with any of the evaluated apps (to minimize bias).

Exclusion Criteria:

- Individuals with visual or motor impairments that could affect app interaction.
- Current or prior employment in app development, usability research, or digital health sectors.

#### 4.3.2. Expert Evaluators

In addition to user testing, a separate group of five expert evaluators participated in the heuristic evaluation phase. According to Nielsen’s guidance, involving 3 to 5 experts is considered optimal for identifying the majority of usability issues [46]. The selected evaluators held professional backgrounds in information technology and demonstrated relevant experience in usability testing, AI-based mobile applications, and healthcare systems, in accordance with established recommendations for heuristic evaluation [47].

### 4.4. The Evaluation Process

To meet the study’s goal, expert and user evaluations were performed using the dual-method approach. The apps were evaluated by five raters separately employing the 13 proposed heuristics of Table 2, both qualitatively and severity-wise. The qualitative results and severity scores were given by the evaluators. A structured scale in the range of 0 (no issue) to 4 (usability catastrophe) was used to rate the severity of each problem [48] by considering its frequency, impact, and persistence, and it is presented in Table 3.

Issues of user interface consistency, transparency, and AI feature reliability are among the subjects that were addressed in their study. It was at the same time that user testing sessions were carried out, and the users were assigned five main tasks that can be seen in Table 4. Every session monitored four main indicators: task success, time on task, number of errors, and satisfaction level. They were measured using the success scale [26]. Errors resulting from different actions than intended [49] were taken as errors and satisfaction was evaluated by the System Usability Scale (SUS).

The performance of each of the participants was investigated on the basis of such metrics as the following:

- Task Success: This was measured in terms of the successfulness of the tasks. A score of 1 stands for a task that is well completed from the first time, 0.5 for tasks that are partially performed (e.g., did with the assistance or did in more than one trial), and 0 for tasks not completed [26].
- Time on Task: The duration was determined by a manual recording using a stopwatch.
- Number of Errors: Unintentional actions that are the cause of errors were taken into account, but system errors were not [49].
- Satisfaction: To measure satisfaction, the System Usability Scale (SUS), a 10-item questionnaire with responses rated on a 5-point Likert scale, was used [49].

Testing occurred in distraction-free environments with reliable internet access. Tools such as Zoom and Excel supported coordination and data collection. Smartphones, tablets, and headsets were used to simulate realistic conditions and ensure audio clarity during remote sessions.

### 4.5. Pilot Validation, Analysis, and Outcomes

A pilot study was conducted to validate the evaluation procedures [50]. Experts reviewed the heuristics (Table 2), and a user test identified some tasks as unclear. The essential refinements were conducted. Data from the qualitative and quantitative methodologies were analyzed. The variable checklists generated qualitative-oriented data, while user testing gathered the numerical information (errors, task success, time). The analysis was extensive and contributed to the emergence of concrete solutions in terms of design. The purpose of the study was to point out the flaws of AI-run mHealth apps and to provide the audience with visual, straightforward, and effective drawings to be used in the future. The results were anticipated to put AI as a competent tool in the healthcare system and bring more strength to the user experience.

## 5. Results

This section presents the results of both expert-based and user-based evaluations of three AI-powered mHealth applications: ADA, Mediktor, and WebMD. It includes findings from heuristic assessments by domain experts and usability testing with participants, covering metrics such as usability problems, task success, completion time, errors, and user satisfaction.

### 5.1. Overview of Selected Applications and Pilot Study

Three AI-powered mHealth applications have been chosen based on the set criteria. These are ADA, Mediktor, and WebMD. ADA is a tool that is powered by AI to gain healthcare and symptom assessments [43]. It definitely combines human knowledge and smart technology and thus makes it possible for the person concerned to access care and get the right medical help [43]. Mediktor is the name of an app that can work with AI and is used for symptom assessment and the navigation of care [45]. WebMD, in addition to being one of the most popular health consultation websites, is also the most reliable health partner of people who want to receive health and disease information and who want to be involved in the decision-making of their health [44]. The pilot study was instrumental in ensuring that the evaluation design and process were sound, effective, and capable of leading to significant learning. Two sets of activities were employed in the research. An expert was requested via email to go through the heuristic evaluation method. After five days, a reply came, indicating the need for the definitions of heuristics and the provision of more explicit examples to support expert insights, as well as the adjustment of some heuristics to better facilitate assessment. User testing was performed with a participant who was directed to complete the evaluation tasks in order to identify some of the possible difficulties such as unclear instructions or wrong task flow. After feedback from the expert and user, some improvements were made that reflected the clarity, coverage, and applicability of each heuristic and the task instructions.

### 5.2. Expert Evaluation Based on the Customized Heuristics

Four experts were selected based on predefined criteria and invited via email to participate in the evaluation. Each expert received two documents: an evaluation guide outlining study objectives and task instructions, and a separate file detailing the customized heuristics. The experts were given seven days to assess the selected applications. The expert evaluation covered three mHealth AI-powered applications—ADA, Mediktor, and WebMD—using the proposed heuristics. Each expert assessed usability issues and assigned severity ratings from 0 to 4.

Table 5 groups and summarizes the 32 usability problems identified in ADA, clustering similar issues under thematic categories to improve readability while preserving the original insights.

The findings in Table 5 highlight several recurring challenges in ADA’s user interface, particularly in areas related to AI explainability, error prevention, and multilingual support. The presence of high-severity issues in AI transparency (H11), user expectation setting (H12), and the accuracy of automation (H13) suggests that users may struggle to understand or trust AI-generated health insights. Additionally, critical gaps in accessibility, personalization, and user-assistance mechanisms indicate that significant usability improvements are needed to effectively accommodate a wider range of users.

Table 6 presents the expert evaluation of Mediktor, grouping 29 identified usability issues under thematic categories to maintain reporting consistency.

The expert evaluations for Mediktor reveal moderate to severe issues in terms of AI communication and user interaction flow. The most critical problems revolve around inadequate explainability and insufficient personalization options for users with varying needs.

Table 7 summarizes 38 usability issues in WebMD by grouping related issues under clear themes for comparative clarity.

The expert evaluation for WebMD highlights numerous usability concerns, especially in areas involving AI transparency, diagnostic accuracy, and content clarity. Severe issues were found in AI explainability and user support, with critical gaps in how the system presents information and guides users through decision-making. These findings suggest that WebMD, despite its widespread recognition, may present risks to users due to opaque system logic, inconsistent navigation, and limited customization options.

### 5.3. Comparative Severity Ratings by Heuristic Category

**Figure 2:**
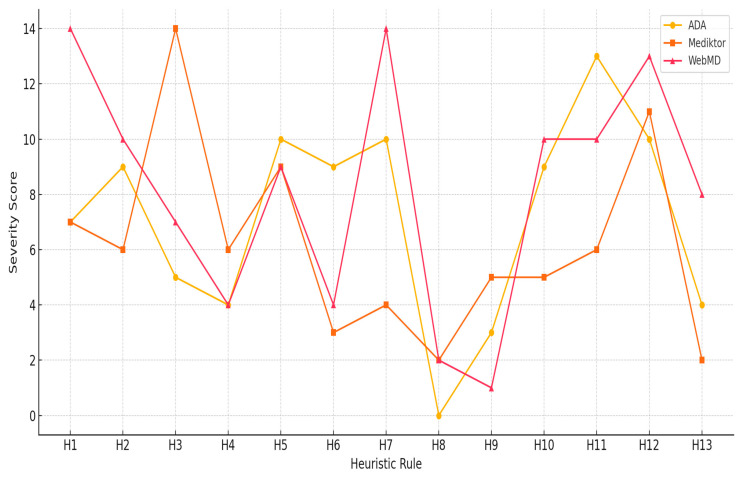
Severity ratings by heuristic for ADA, Mediktor, and WebMD.

Examining the entire situation thoroughly for usability concerns in these mHealth applications powered by AI could be better understood by conducting a comparative analysis based on the severity scores for the 13 heuristic categories in each application, the experts, and ADA, Mediktor, and WebMD. In the evaluation, every heuristic problem was rated on a scale of 0 (no) to 4 (usability catastrophe). The total scores were determined by the respective frequency of the severity level multiplied by the grade. Figure 2 illustrates the cumulative severity ratings for each device across the applications.

Figure 2 illustrates notable distinctions in heuristic severity across the three evaluated mHealth applications. ADA demonstrated the highest severity scores in Transparency and Explainability (H11) and User Expectations (H12), indicating substantial challenges related to AI communication and user trust. Mediktor, on the other hand, revealed critical issues in User Control and Freedom (H3) and Error Prevention (H5), reflecting concerns with navigation and input validation. WebMD displayed consistently high severity in AI Explainability (H11–H13) and Recognition and Recall (H6), suggesting deficiencies in conveying automation processes and supporting personalized interactions. This comparative visualization provides a clearer understanding of the most problematic heuristics in each application and informs prioritization for future design improvements.

### 5.4. User Evaluation Based on the Selected Applications

To evaluates usability from an end-user perspective, a user-based evaluation was conducted on the three selected AI-powered mHealth applications: ADA, Mediktor, and WebMD. Twelve participants with varying backgrounds, age groups, and device experience levels were recruited and observed while completing a standardized set of five core tasks. Their performance was documented across four key metrics: task success, task completion time, number of errors, and overall satisfaction.

#### 5.4.1. Task Completion Success Rates

Each participant was instructed to complete five predefined tasks on each application. Task success was recorded as 1 for successful completion on the first attempt, 0.5 for partial completion (e.g., completed with assistance or multiple attempts), and 0 for failure to complete the task. The average success score for each task was then calculated per application to determine the ease of task completion and identify areas where users faced difficulty.

**Figure 3:**
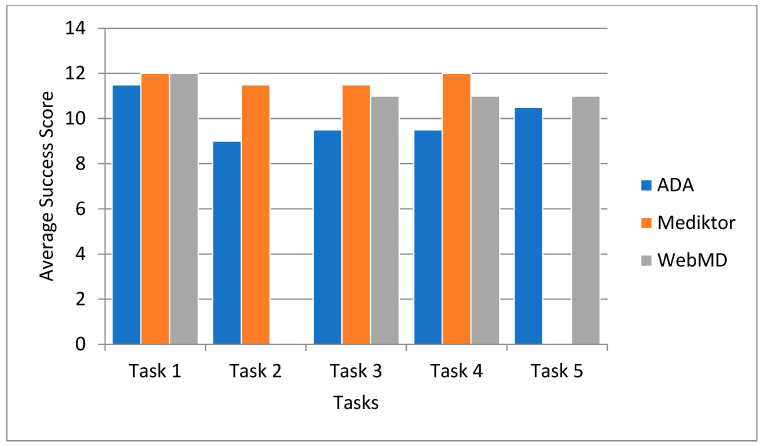
Average task success rates across ADA, Mediktor, and WebMD.

Figure 3 explores the average task success rates across the three applications—ADA, Mediktor, and WebMD—based on the cumulative performance of all participants in each task.

Figure 3 illustrates the comparative task success rates across the ADA, Mediktor, and WebMD applications. ADA and Mediktor demonstrate high average success rates across most tasks. WebMD exhibits comparable performance except for Task 2, which was unsupported across participant devices, resulting in a data gap.

#### 5.4.2. Task Completion Time

**Figure 4:**
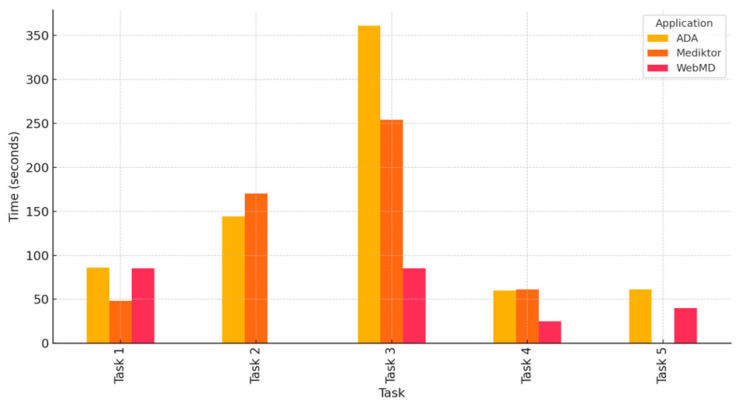
Average task completion time across ADA, Mediktor, and WebMD.

To understand user efficiency, the average time to complete each task was calculated for each application. Task duration serves as a metric of both usability and complexity. Figure 4 introduces the average time (in seconds) spent per task, indicating efficiency and complexity for each application.

Figure 4 shows the average time in seconds for each task across the three applications. ADA demonstrated consistent times, while Mediktor showed longer durations for Task 2 and Task 3. WebMD had the shortest durations overall but again lacked data for Task 2.

#### 5.4.3. Number of Errors

**Figure 5:**
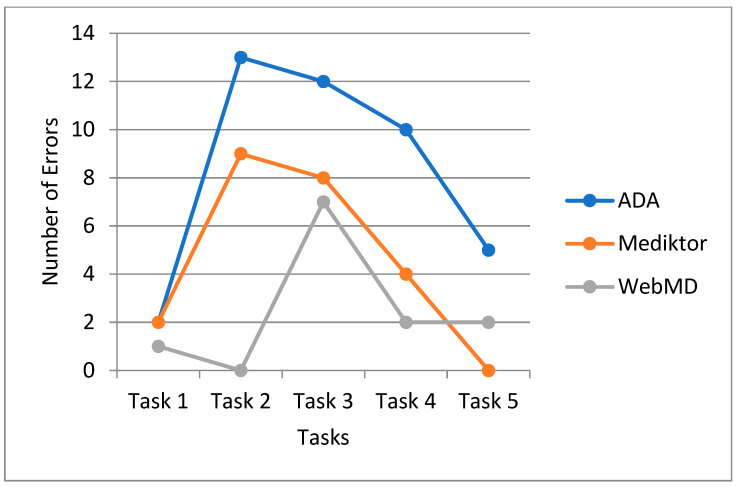
Total number of user errors across ADA, Mediktor, and WebMD.

Errors represent usability breakdowns where users perform unintended actions. Each observed mistake was counted per task and application. Figure 5 introduces the total number of errors observed during task execution per application.

Figure 5 depicts the number of errors users made while performing tasks on each app. ADA users committed the highest number of errors in Task 2 and Task 3. Mediktor showed fewer errors overall, while WebMD had moderate errors with fewer in later tasks.

#### 5.4.4. Overall Satisfaction (SUS Score)

**Figure 6:**
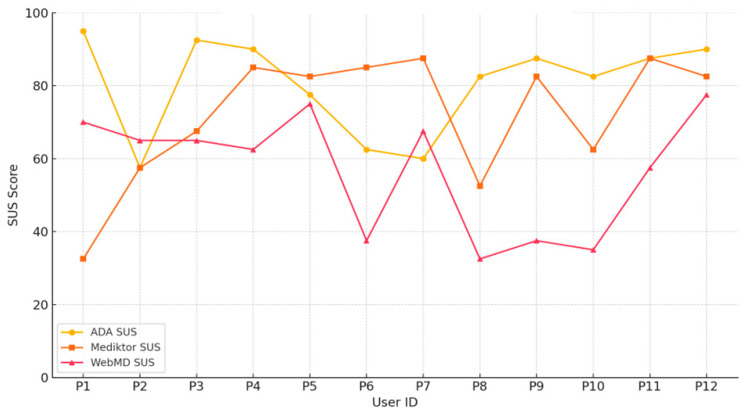
System Usability Scale (SUS) scores per user across ADA, Mediktor, and WebMD.

User satisfaction was assessed using the System Usability Scale (SUS), where each participant rated their experience with each application. The SUS score ranges from 0 to 100, with higher scores indicating better perceived usability. Figure 6 below displays individual user scores across the three applications.

Figure 6 demonstrates that ADA received the highest average satisfaction score (80.4), followed by Mediktor (72.0), while WebMD trailed behind with an average score of (56.8), reflecting a comparatively lower perceived usability.

### 5.5. Inferential Statistical Analysis

To validate the usability differences observed in the descriptive results, inferential statistics were applied using data collected from 30 participants. This sample size satisfies the minimum requirement for parametric testing and ensures adequate statistical power for within-subject comparisons.

Before performing the inferential tests, the normality assumption required for repeated-measures ANOVA was assessed using the Shapiro–Wilk test:

- ADA: W = 0.975, *p* = 0.687;
- Mediktor: W = 0.984, *p* = 0.913;
- WebMD: W = 0.963, *p* = 0.365.

All *p*-values were above 0.05, indicating that the SUS scores for each application are approximately normally distributed. This justifies the use of parametric statistical methods. Since the same participants evaluated all three applications, a repeated-measures ANOVA was conducted to compare user satisfaction (SUS scores) across the applications. This within-subjects approach aligns with the study design and appropriately accounts for the dependence between measures. To explore specific differences between pairs of applications, paired-sample *t*-tests were conducted.

**Figure 7:**
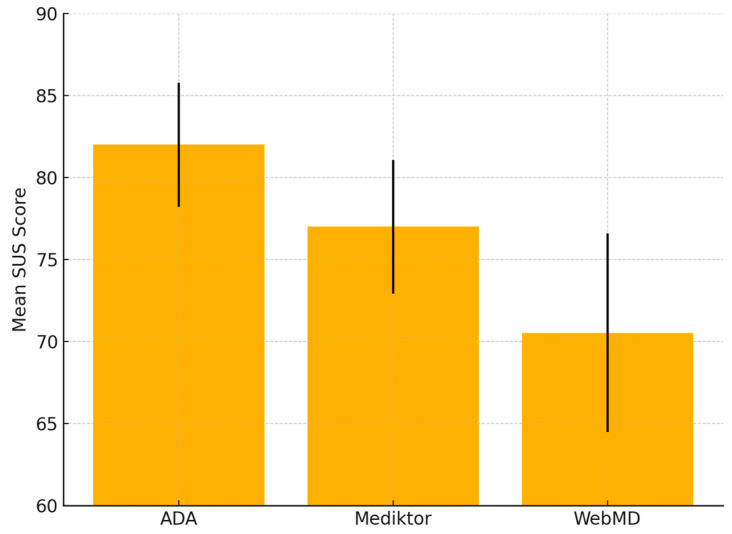
SUS scores (N = 30) with standard deviations for ADA, Mediktor, and WebMD.

Figure 7 illustrates the mean SUS scores across ADA, Mediktor, and WebMD, with error bars representing the standard deviations, highlighting differences in user satisfaction.

As shown in Figure 7, ADA achieved the highest mean SUS score (M = 81.29), followed by Mediktor (M = 76.51), and WebMD (M = 70.61). The standard deviations are visually represented as error bars.

- Repeated-Measures ANOVA Results:
  ○ F(2, 58) = 27.49, *p* < 0.001, partial η^2^ = 0.487.
- Post Hoc Paired Sample *t*-Test Results:
  ○ ADA vs. WebMD: t(29) = 7.34, *p* < 0.001;
  ○ ADA vs. Mediktor: t(29) = 3.94, *p* = 0.0004;
  ○ Mediktor vs. WebMD: t(29) = 3.71, *p* = 0.0009.

These results indicate statistically significant differences among the three applications, thereby supporting the descriptive findings presented earlier. ADA demonstrated the highest level of user satisfaction, followed by Mediktor, with WebMD receiving the lowest scores. The inclusion of data from 30 participants enhances the statistical power and robustness of the analysis. Overall, the inferential findings corroborate and strengthen the study’s conclusions, aligning consistently with the usability patterns observed throughout the evaluation. This evidence reinforces the study’s conclusions about relative usability among AI-powered mHealth applications.

## 6. Discussion

This study evaluated the usability of three AI-powered mHealth applications by triangulating expert heuristic reviews, real-user testing, and brief automated checks. Integrating findings from these complementary methods provides nuanced insight into each app’s strengths and weaknesses.

### 6.1. Integration of Expert and User Results

Expert heuristic evaluations generally rated the apps well on traditional principles such as consistency and visual design, while user testing uncovered deeper concerns related to transparency, task flow, and error handling. ADA earned the highest expert ratings and the top System Usability Scale score, yet multi-symptom tasks such as chest discomfort exposed error-handling limitations, illustrating a mismatch between expert prediction and user performance. Across all apps, experts noted missing transparency cues, such as the absence of confidence scores or explanations of symptom-checker decisions [26,49]. Users echoed this concern during follow-up tasks, often hesitating or misinterpreting outputs. Wilcoxon signed-rank tests confirmed statistically significant gaps between expert expectations and user performance on Tasks 3 and 4. Mediktor conformed adequately to heuristics but several users struggled with its branching question logic. WebMD, despite strong brand recognition, scored lowest in both expert and user assessments because of a cluttered interface and limited interactive feedback. This convergence underscores critical design gaps in popular AI-enabled mHealth tools. These results show that heuristic reviews can predict many interface issues but may underrepresent real-world complexities such as multi-step reasoning and variable user health literacy. Combining expert and user perspectives therefore yields a more robust understanding of usability.

### 6.2. Explainability and Trust Gaps

Explainability remained a major weakness in all evaluated apps. None of the systems provided a rationale for diagnoses, confidence levels, or links to clinical guidelines. This lack of transparency limits user trust, a pivotal factor in healthcare contexts [7,29,30]. Although ADA occasionally presented follow-up advice, it lacked specific justifications. Users consistently requested clearer AI explanations and more control over the information shown. Suggestions included optional detail toggles and summaries of how inputs influenced results, mirroring calls in the explainable AI literature.

### 6.3. Implications for Design and Evaluation

The effective design of AI-powered mHealth apps must go beyond traditional heuristics and include real-world testing of explainability and error handling. The dual-layer evaluation used in this study uncovered blind spots that a single method might miss. Design teams should adopt progressive disclosure for complex outputs, present confidence indicators, and make decision logic traceable. Integration with electronic health records and body-sensor data can improve personalization and diagnostic accuracy [51,52]. Consistent terminology and clear error messages will further support user understanding and trust [53]. Developers should also provide accessible help resources such as tutorials, FAQs, or chatbots, allow users to review and edit past assessments, and offer interface customization options. Multilingual support, including Arabic, and accommodations for visual accessibility will broaden inclusivity. Finally, incorporating natural-language interactions and links to validated medical sources can strengthen credibility and user engagement.

Ultimately, the triangulated findings of this study demonstrate that AI-enabled mHealth applications continue to face critical challenges in transparency, flexible task flows, and user-centered error prevention. Addressing these issues requires integrating expert and user evaluations alongside AI-specific usability heuristics. Future research should explore larger and more diverse user samples, as well as long-term user engagement, to assess how trust and usability evolve over time in real-world settings.

## 7. Conclusions

This study conducted a triangulated usability evaluation of three AI-powered mHealth applications—ADA, Mediktor, and WebMD—using heuristic analysis, user testing, and automated inspection. While ADA demonstrated relatively higher usability in terms of SUS scores and task success, all apps showed critical shortcomings in transparency, user guidance, and explainability features. The findings revealed that users encountered significant navigational and input-related difficulties, especially in WebMD. Moreover, none of the apps presented confidence scores, rationale explanations, or robust feedback mechanisms, indicating low compliance with transparency and explainable AI (XAI) principles. Although heuristic and user evaluations identified consistent usability trends, these findings are limited by the small sample size and the descriptive nature of some metrics. As such, our conclusions are intended to highlight areas of improvement rather than assert definitive superiority among the apps. Future work should involve larger and more diverse user samples, as well as deeper evaluation of clinical accuracy and long-term user engagement. Designers and developers are encouraged to adopt XAI principles, ensure interface consistency, and enhance feedback systems to improve trust and usability in AI-driven healthcare applications.

## Figures and Tables

**Table 1:** Nielsen’s usability heuristics.

Heuristic Usability Rule	Concise Definition
H1: Visibility of system status	Keep users informed with timely and clear feedback.
H2: Match with real world	Use user-friendly language and follow real-world conventions.
H3: User control and freedom	Allow easy undo/redo and exit from unintended actions.
H4: Consistency and standards	Follow platform and user interface conventions consistently.
H5: Error prevention	Prevent errors through clear instructions and validations.
H6: Recognition over recall	Make elements visible to reduce memory load.
H7: Flexibility and efficiency of use	Support customization and shortcuts for frequent users.
H8: Aesthetic and minimalist design	Present only relevant and essential information.
H9: Help with errors	Use clear, helpful messages to explain and resolve errors.
H10: Help and documentation	Provide accessible guidance or help when needed.

**Table 2:** Proposed heuristics evaluation [23,38,39,40,41].

Heuristic Usability Rule	Definition
H1: Visibility of system status	Keep users informed with timely and appropriate feedback.
H2: Match between system and real world	Use user-friendly language and follow real-world conventions.
H3: User control and freedom	Support undo/redo and allow exiting unwanted actions easily.
H4: Consistency and standards	Follow familiar conventions and consistent behaviors.
H5: Error prevention	Prevent errors through clear design and proactive alerts.
H6: Recognition rather than recall	Make options visible to reduce users’ memory load.
H7: Flexibility and efficiency of use	Allow customization and efficient workflows.
H8: Aesthetic and minimalist design	Keep interface clean and free from unnecessary content.
H9: Help users recognize, diagnose, and recover from error	Provide plain-language error messages with solutions.
H10: Help and documentation	Offer accessible documentation when needed.
H11: Transparency and explainability	Explain how AI generates its insights or decisions.
H12: User’s expectations	Clarify what AI can and cannot do.
H13: Accuracy and reliability of automation and AI	Ensure reliable AI using quality data and contextual checks.

**Table 3:** Severity rating scale [48].

Rating Number	Description
0	I don’t agree that this is a usability issue
1	Superficial usability issue
2	Minor usability issue
3	Major usability issue
4	Usability catastrophe

**Table 4:** List of Evaluated Usability Tasks [48].

No.	Task
1	Create a new account.
2	Update your personal profile with three medical information.
3	Start a new symptom-checking assessment using “back pain,” “cough,” or “fever.”
4	Search for symptoms like “anxiety,” “panic attack,” or “cold.”
5	Track new symptoms by specifying the date and time.

**Table 5:** Thematic summary of expert evaluation (ADA).

Theme	Representative Problem	Violated Heuristic(s)	Severity Rating(s)
Error Message Clarity	Vague error for email format	H9	3
Language Support	No Arabic language support	H2, H7	3, 4
Input Validation and Error Prevention	Invalid inputs allowed	H5	4, 3, 3
Inconsistent Design and Navigation	Button style and layout inconsistencies	H4	2
Feedback and Loading	No progress indicators	H1	1, 4, 2
Navigation and Undo	No undo/backtrack options	H3, H6	2, 3, 2, 4
Accessibility and Visual Design	Low contrast, cluttered UI	H7, H8, H6	3, 0, 1, 2
AI Explainability	No clarity on AI outputs	H11, H12, H13	4, 4, 4
Help and Documentation	Help/tips missing or buried	H10	3, 3
Terminology and Jargon	Unclear medical terms	H2	4
Personalization	No saved settings or custom options	H7	3
Memory Load	No history or symptom recall	H6	1, 4
AI Limitations	AI scope not explained	H12	4, 4, 2
Redundancy and Layout	Confusing button placement	H2, H4	2
Educational Resources	No AI or onboarding guidance	H12	2

**Table 6:** Thematic summary of expert evaluation (Mediktor).

Theme	Representative Problem	Violated Heuristic(s)	Severity Rating(s)
Language and Terminology	Misleading terms; unclear jargon	H2	3
Accessibility and Visual Design	No text scaling; poor contrast/icons	H7, H1	3, 1, 1
Error Prevention	No input validation or corrections	H5	3, 4
Inconsistent Navigation	Poor undo/redo; inconsistent layout	H3, H4	4, 2
AI Explainability	No guidance or transparency in AI	H11, H12, H13	3, 4, 2
Help and Documentation	Help missing or hard to find	H10	3, 2
Feedback and System Status	No system messages or indicators	H1, H9	2, 2
Redundancy and Layout Confusion	Duplicate features under new names	H4	2

**Table 7:** Thematic summary of expert evaluation (WebMD).

Theme	Representative Problem	Violated Heuristic(s)	Severity Rating(s)
Language and Terminology	Unclear medical terms; lacks Arabic support	H2, H7	3, 4
Error Messaging	Vague or missing error messages; lack of warnings	H5, H9	4, 3, 1
Navigation and Undo	Limited backtracking; account deletion difficult	H3	2, 3
AI Explainability	No info on AI-generated results or model limitations	H11, H12, H13	4, 4, 4
Visual and Interface Design	Crowded UI, poor content segmentation, inconsistent design	H8, H4, H6	2, 2, 4
Help and Documentation	Help section buried; limited tutorials or guidance	H10	3, 4
Personalization and Recall	Lack of saved searches, no dashboard customization	H7, H6	3, 4
Diagnosis and Input Gaps	Limited input flexibility; potential misdiagnosis risks	H5, H12	4, 3
Transparency and Data Sources	No data source disclosures; vague metric indicators	H11	3

## Data Availability

The data presented in this study are available on request from the corresponding author. The data are not publicly available due to privacy or institutional restrictions.
